# Potential effect of luteolin, epiafzelechin, and albigenin on rats under cadmium-induced inflammatory insult: *In silico* and *in vivo* approach

**DOI:** 10.3389/fchem.2023.1036478

**Published:** 2023-03-01

**Authors:** Andleeb Shahzadi, Nusrat Tariq, Haktan Sonmez, Sulayman Waquar, Ayesha Zahid, Muhammad Ahsan Javed, Muhammad Yasin Ashraf, Arif Malik, Munir Ozturk

**Affiliations:** ^1^ Department of Medical Pharmacology, Cerrahpasa Medical Faculty, Istanbul University-Cerrahpasa, Istanbul, Turkiye; ^2^ Department of Physiology, M. Islam Medical and Dental College, Gujranwala, Pakistan; ^3^ School of Biochemistry, Minhaj University Lahore, Lahore, Pakistan; ^4^ Institute of Molecular Biology and Biotechnology (IMBB), The University of Lahore, Lahore, Pakistan; ^5^ School of Pharmacy, Minhaj University Lahore, Lahore, Pakistan; ^6^ Centre for Environmental Studies, Ege University, Izmir, Turkiye

**Keywords:** docking studies, antioxidant, COX-2 inhibitor, anti-inflammatory, AutoDock Vina

## Abstract

**Introduction:** Cadmium(Cd) an industrial poison present abundantly in the environment, causes human toxicity by an inflammatory process. Chronic exposure of cadmium can cause a number of molecular lesions that could be relevant to oncogenesis, through indirect or epigenetic mechanisms, potentially including abnormal activation of oncogenes and suppression of apoptosis by depletion of antioxidants. As induction of cyclooxygenase (COX)-2 is linked to inflammatory processes, use of luteolin, epiafzelechin, and albigenin alone or in different combinations may be used as anti-inflammatory therapeutic agents.

**Methods:** We, herein, performed in silico experiments to check the binding affinity of phytochemicals and their therapeutic effect against COX-2 in cadmium administered rats. Wistar albino rats were given phytochemicals in different combinations to check their anti-inflammatory activities against cadmium intoxication. The level of alanine aminotransferases (ALT), 4-hydroxynonenal (4HNE), 8-hydroxy-2-deoxyguanosine (8-OHdG), tumor necrosis factor-alpha (TNF-α), isoprostanes (IsoP-2α), COX-2, and malondialdehyde (MDA) were estimated with their respective ELISA and spectrophotometric methods.

**Results:** The generated results show that phytocompounds possessed good binding energy potential against COX-2, and common interactive behavior was observed in all docking studies. Moreover, the level of ALT, 4HNE, 8-OHdG, TNF-α, IsoP-2α, malondialdehyde, and COX-2 were significantly increased in rats with induced toxicity compared to the control group, whereas in combinational therapy of phytocompounds, the levels were significantly decreased in the group.

**Discussion: **Taken together, luteolin, epiafzelechin, and albigenin can be used as anti-inflammatory therapeutic agents for future novel drug design, and thus it may have therapeutic importance against cadmium toxicity.

## 1 Highlights

• AutoDock Vina program was used to perform molecular docking of *luteolin*, *epiafzelechin*, and *albigenin* with COX-2.
• Wistar albino rats were given phytochemicals to evaluate the anti-inflammatory effects against cadmium-induced insult.
• Luteolin, epiafzelechin, and albigenin can be used as anti-inflammatory agents for future novel drug designing.

## 2 Introduction

Chronic exposure to heavy metals like cadmium (Cd) can cause a number of molecular lesions that could be relevant to oncogenesis, through indirect or epigenetic mechanisms, potentially including abnormal activation of oncogenes and suppression of apoptosis (Waalkes, 2003). Cadmium intoxication results in the depletion of antioxidants such as glutathione, superoxide dismutase, and catalase that damage the lipid content of the cell membrane and induce lipid peroxidation and oxidative stress (Valko et al., 2005).

About 600 metric tons of Cd is synthesized in the United States annually, of which 150 metric tons is inhaled (Nordberg et al., 2007). One of the major factors involved in Cd toxicity is cigarette smoking, and there are high levels of Cd present in the blood of smokers (Richter et al., 2017). Other sources of Cd exposure include unhygienic food, water (that is present in sealed water pipes), drugs, and various dietary supplements (Abernethy et al., 2010). Cd, an industrial poison present abundantly in the environment because of slow processes of destruction and abrasion of rocks and soil, particularly due to fire in the forest and volcanic explosion, leads to an increase in environmental Cd (Everett and Frithsen, 2008). Animals and plants are highly exposed to environmental Cd, which is major cause of human Cd toxicity that destroys various biological organs, including the lungs, liver, kidney, bones, testes, and placenta (Bertin and Averbeck, 2006; Pari and Murugavel, 2007).

Bioinformatics is an exciting area that utilizes mathematical, statistical, and computational approaches for biological problems. The prediction of protein structures by NMR and X-ray crystallography techniques consumes time (Magana et al., 2014)*. In silico* studies utilized various tools for model building of the targeted protein and molecular docking which are less time-consuming. The homology modelling approach was used to build a 3D structure of the targeted protein (Schmidt et al., 2014). The 3D structure of COX-2 is not reported in the Protein Data Bank. In this current study, *in silico* techniques are utilized to predict the 3D structure of COX-2 by homology modelling approaches to reveal the insight into COX-2 3D structure. The 3D structure of the targeted protein is fundamental for computational drug design.

Nature provides ample resources that are valuable to humankind. The selection of phytochemicals is based on their significance and novelty. The literature depicts the analyses of various phytochemicals; however, their solo and synergistic effects among the disease models need to be illustrated. *Acacia senegal* is found abundantly in Eastern Africa from Mozambique to South Africa and in tropical areas of Western and Central Africa (Odee et al., 2012). First, it was discovered in Australia, Egypt, the Virgin Islands, Puerto Rico, and South Asia. *Acacia senegal* is mostly cultivated in Nigeria, Pakistan, and India. It consists of various phytochemicals, playing a major role in food (flavor fixative, emulsifier, and stabilizer of dairy products), pharmaceutics, and therapeutics. Among its various phytocompounds, luteolin is a naturally occurring flavone available with significant biological properties (Muruganathan et al., 2022), limited to not only ameliorating breast cancer (Cook, 2018) but also attenuating sepsis-induced myocardial injury by enhancing autophagy in mice (Wu et al., 2020) and playing a significant hepatoprotective role (Balamurugan and Karthikeyan, 2012; Zhang et al., 2017).

*Albizia lebbeck* is commonly cultivated in Northern Australia, Africa and Asia regions and *Acacia chundra* is a perennial plant commonly found in the Asia region (Lampe et al., 2008). The two phytocompounds albigenin and epiafzelechin (present in *Albizia lebbeck* and *Acacia chundra*, respectively) exhibited therapeutic application in various inflammatory conditions (Lakshmi et al., 2018). Albigenin extract is derived from albigenic acid derived from the bark of *Albizia lebbeck Benth* (Barua and Raman, 1959). *Albizia lebbeck* (Family: Mimosaceae) possesses medicinal properties and has been conventionally used to treat several conditions, such as convulsions (Kasture et al., 2000) and anxiety, and to enhance the cognitive functions (Une et al., 2001).

Similarly, epiafzelechin is a flavonoid found in nature and is reported to be extracted from the leaves of *E. acoroides.* The extract is a white crystalline powder with antioxidant properties that inhibit oxidative radicals’ scavenging activity. It has been reported to inhibit lipid peroxidation responsible for increasing DNA damage (Seccon et al., 2010). Recent studies showed its protective effects against bone loss due to ovariectomy in adult mice (Wong et al., 2017). In another study, epiafzelechin prevented SARS-CoV-2 replication or transcription by locking 3CL^pro^ and RdRp viral proteins (Dey et al., 2021).

Hence, the current study examines the synergistic use of the phytocompound (luteolin, albigenin, and epiafzelechin) extract to evaluate and determine the effects when administered alone or in combination on the Cd-induced signaling cascade involved in inflammation and DNA damage. We also aim to investigate the new dimensions for novel drug designing by targeting COX-2 through *in silico* studies.

## 3 Materials and methods

### 3.1 Protein structure prediction

The amino acid sequence (604 residues) of COX-2 protein was obtained from UniProt with accession number P35355 in the FASTA format (https://www.uniprot.org/uniprot/P35355). To predict the protein model, BLASTp was used against the Protein Data Bank (PDB) to search for the most appropriate template with the query sequence. The template was selected based on sequence identity and similarity, query coverage, and E-value. Finally, the MODELLER v9.17 tool was used to build the protein model by using a couple of commands: align2d.py and get-model.py (Eswar et al., 2008). The structural assessment of the generated model was confirmed through multiple online tools like PROCHECK (Laskowski et al., 1993), ERRAT (Colovos and Yeates, 1993), VERIFY3D (Eisenberg et al., 1997), and ANOLEA (Melo and Feytmans, 1998). The selected three-dimensional (3D) model of COX-2 was further minimized using UCSF Chimera v1.12 (Meng et al., 2006) at 1,000 steepest and 1,000 conjugate gradient runs with AMBER force field parameters.

### 3.2 Phytochemical preparation and molecular docking

Based on the literature survey, three phytocompounds, luteolin, albigenin, and epiafzelechin, were retrieved from PubChem (Bolton et al., 2008). The selected phytocompounds were sketched in ChemDraw Ultra and retrieved in the PDB format. For the comparative study, a standard drug (clavazin) was selected. The energy minimization of selected three phytocompounds was minimized using UCSF Chimera. The docking binding pocket of the target protein was predicted using CASTp (Dundas et al., 2006) and 3DLigandSite before energy minimization (Wass et al., 2010). AutoDock Vina was utilized for molecular docking (Trott and Olson, 2010). Drug properties of selected phytocompounds were calculated. Mcule (Kiss et al., 2012) and ADMET profile were calculated using the admetSAR online server (Cheng et al., 2012). UCSF Chimera was used to analyze the results of molecular docking.

### 3.3 Ethical approval

The experiment was performed in animal house and biochemical analysis was conducted at the Institute of Molecular Biology and Biotechnology (IMBB) of the University of Lahore. The experiment was approved and performed according to the guidelines of the Ethical Research Committee of the University of Lahore.

### 3.4 Plant extract

The standardized extract of phytochemicals luteolin (Product ID: 72511), epiafzelechin (Product ID: FE139053), and albigenin (Product ID: FDB015558) was purchased separately from Sigma-Aldrich Corporation (St. Louis, MO, United States), Biosynth (United States), and Genome Canada, respectively, and later their different doses were prepared according to the study design.

### 3.5 Study designing

The Wister albino rats were divided randomly into nine groups. Group A was the control group without inducing hepatotoxicity with Cd exposure. Rats in group B were induced hepatotoxicity with exposure to Cd 1.5 mM/kg body wt/d (90 days). Rats in groups C, D, and E were induced hepatotoxicity with exposure to Cd and also treated with single therapy of luteolin, epiafzelechin, and albigenin (@500 mg/kg body wt.), respectively. A preliminary study was carried out to check the toxicity of oral dosage of selected phytochemicals (luteolin, epiafzelechin, and albigenin) in the rat model, and guidelines for the subacute toxicity were followed. A single dose of 500 mg/kg administered orally to albino rats was studied for acute toxicity. Independent doses of 100, 200, 400, and 500 mg/kg were administered orally for 28 consecutive days, and findings for each parameter were then compared with healthy ones. No significant change in weight of selected organs was reported compared to healthy ones. No major toxicity or organ damage was reported, and the respective dose was considered to be safe for the administration in animal models. Moreover, the results were verified with the help of RSM (Response Surface Methodology).

Group F was exposed to Cd to induce liver damage and then treated with the combination of luteolin + epiafzelechin. The combination of two phytochemicals luteolin + albigenin was fed to rats exposed to Cd in group G. Group H was induced liver damage with Cd exposure and was administered with the combination of two phytochemicals epiafzelechin + albigenin. Group I animals received the combination therapy of three phytochemicals luteolin + epiafzelechin + albigenin to investigate their effect on liver damage induced by Cd (Table 1).

**TABLE 1:** Experimental design.

Group (*n* = 6)	Treatment
**A**	Control
**B**	Cd alone
**C**	Cd + luteolin
**D**	Cd + epiafzelechin
**E**	Cd + albigenin
**F**	Cd + luteolin + epiafzelechin
**G**	Cd + luteolin + albigenin
**H**	Cd + epiafzelechin + albigenin
**I**	Cd + luteolin + epiafzelechin + albigenin

Dose of Cd (1.5 mmol/kg B. Wt for 3 months).

Dose of luteolin, epiafzelechin, and albigenin (500 mg/kg B. Wt).

### 3.6 Biochemical analysis

A volume of 5 ml of the blood sample was taken from the heart of the Wister albino rats during dissection, and serum was separated from the blood samples after centrifugation for 10 min at 4000 rpm.

#### 3.6.1 Estimation of oxidative and inflammatory biomarkers

The samples were analyzed for the levels of alanine aminotransferase (ALT), malondialdehyde, cyclooxygenase-2 (COX-2), 8-hydroxy-2′-deoxyguanosine (8-OHdG), isoprostanes, 4-hydroxynonenal (4-HNE), and tumor necrosis factor-α (TNF-α) using commercially available ELISA kits (Abcam), following their respective protocols.

### 3.7 Statistical analysis

The data were subjected to analyses of variance using a CoStat computer package (Cohort Software, Berkley, California). The mean values were compared with the least significance difference test following the methods proposed by Snedecor and Cochran. Statistical analysis was performed using one-way analysis of variance (ANOVA) followed by Duncan’s multiple range test (DMR-test). Pearson’s correlation test was used to find the statistical relationship between two variables. Values are represented as mean ± SD, and *p*-values < 0.05 were considered significant.

## 4 Results

### 4.1 Protein structure analysis

**FIGURE 1:**
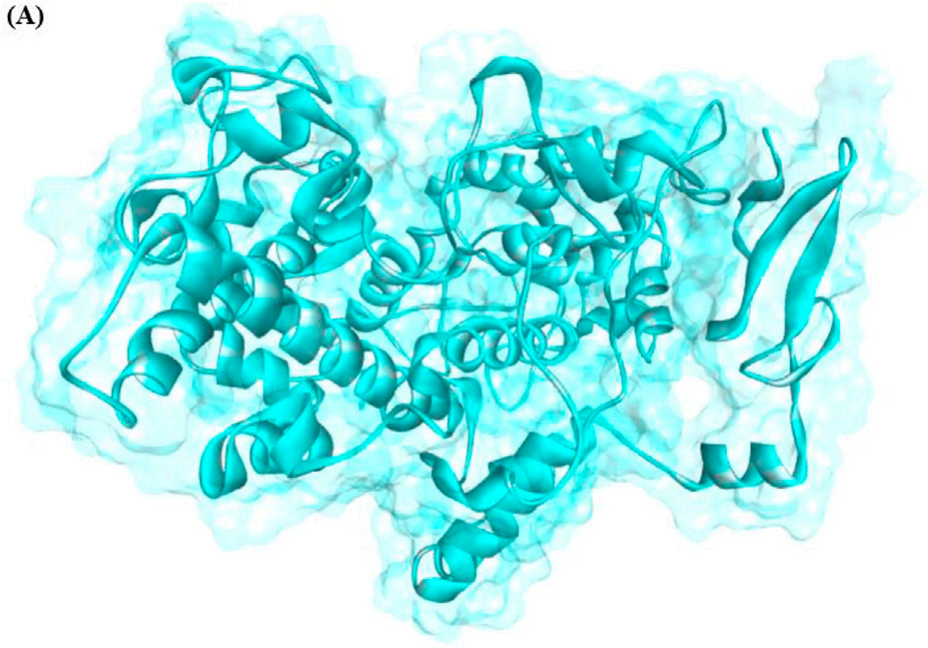
Generated 3D structure of COX-2.

The structure validation of COX-2 was confirmed using ERRAT and VERIFY3D. The predicted results showed that the generated model has good structural accuracy, and most of the residues were present in the allowed regions. The predicted model of the protein is shown in Figure 1.

### 4.2 Chemoinformatic analysis of selected phytochemicals

**FIGURE 2:**
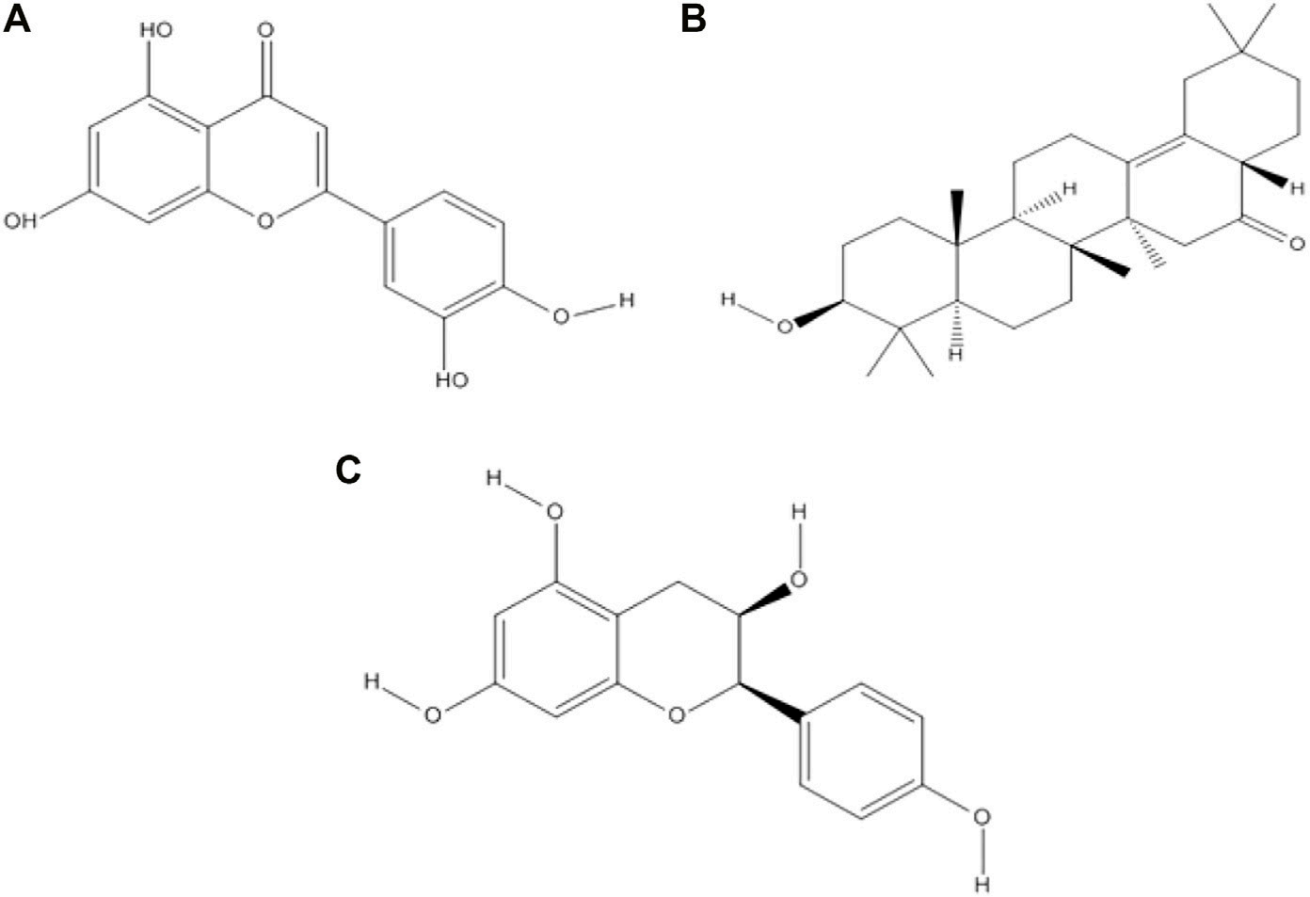
Two-dimensional structures of three selected phytocompounds. **(A)** Luteolin, **(B)** albigenin, and **(C)** epiafzelechin.

Drug-likeness properties were calculated using the Mcule online server (Table 2). There are three phytocompounds luteolin, albigenin, and epiafzelechin examined in this study (Figures 2 A–C). The chemoinformatic analysis showed that all the three phytocompounds possessed good biological properties. The molecular weight of all the three compounds was comparable with the standard value (<500 mg/mol). Moreover, the hydrogen bond acceptor/donor (HBA/D) was less than 10 and 5, respectively. However, the LogP value of albigenin was more significant than 5, while that of both luteolin and epiafzelechin was 2.28 and 1.84, respectively. The Lipinski Rule of Five testified against all three compounds revealed that albigenin showed RO5 violation, whereas luteolin and epiafzelechin obeyed the rule.

**TABLE 2:** Drug properties of selected phytocompounds and standard drug clavazin.

Phytocompound property	Luteolin	Albigenin	Epiafzelechin
Molecular weight (g/mol)	286.23	426.67	274.26
LogP	2.28	7.10	1.84
Hydrogen bond acceptor	6	2	5
Hydrogen bond donor	4	1	4
Rotatable bonds	1	0	1
PSA	111.13	37.30	90.15
RO5 violations	0	1	0
Atoms	31	77	34
Rings	3	5	3

### 4.3 Pharmacokinetic analysis of selected compounds

The ADMET profile of selected phytocompounds was calculated by admetSAR (Table 3). Two parameters were selected for absorption: blood–brain barrier (BBB) penetration and human intestinal absorption (HIA). For BBB penetration, luteolin showed negative results, whereas albigenin and epiafzelechin exhibited positive results. Moreover, positive values (+0.9650, + 0.8704, and + 0.9857) were observed against intestinal absorption, which ensures that all three phytochemicals may potentially cross intestinal barriers through absorption. Moreover, all three compounds exhibited non-AMES and non-carcinogenic behavior. The overall results showed that all three compounds possessed good lead-like behavior based on these ADMET results and can be used for further analysis.

**TABLE 3:** ADMET profile analyses of phytocompounds.

Name of properties	Luteolin	Albigenin	Epiafzelechin
BBB	−0.5711	+0.9382	+0.6559
HIA	+0.9650	+0.8704	+0.9857
CYP inhibitory promiscuity	High CYP	Low CYP	Low CYP
AMES toxicity	Non-AMES toxic	Non-AMES toxic	Non-AMES toxic
Carcinogens	Non-carcinogens	Non-carcinogens	Non-carcinogens
Acute oral toxicity	0.7348	0.8694	0.4540
Aqueous solubility	−2.9994	−4.0877	−3.2332

^a^ BBB, blood–brain barrier; HIA, human intestinal absorption.

### 4.4 Molecular docking

#### 4.4.1 Binding affinity (Kcal/mol)

The molecular docking study was utilized to identify binding pockets and affinities of these compounds against the target protein. Table 4 shows that all three phytochemicals were bound within the active binding sites of the target protein. Luteolin, albigenin, and epiafzelechin exhibited good binding energy values (−10.3, −9.6, and −7.8 kcal/mol, respectively) compared to a standard drug clavazin (−12.5 kcal/mol).

**TABLE 4:** Binding affinities of phytocompounds and clavazin against COX-2.

Docked complex	Binding affinity (Kcal/mol)
Luteolin–COX-2	−10.3
Albigenin–COX-2	−9.6
Epiafzelechin–COX-2	−7.8
Clavazin (glycyrrhizic acid)	−12.5

#### 4.4.2 Binding interactions

**FIGURE 3:**
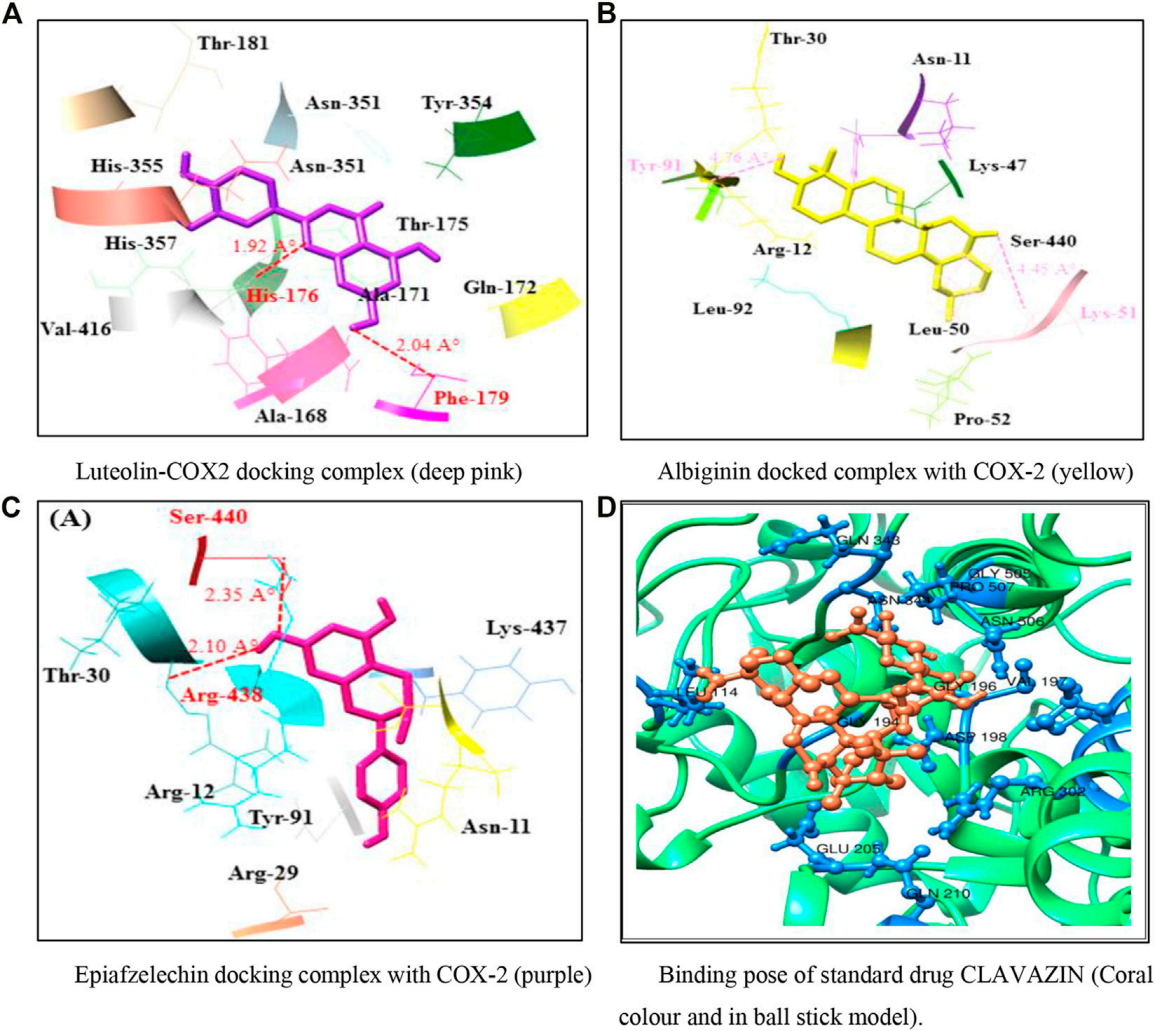
**(A–D)** Docking models representing binding poses of the phytocompounds and standard drug.

Analyzing the docking complex confirmed the binding conformation positions of phytochemicals inside the binding pocket. In the luteolin–COX-2 docking complex, a couple of hydrogen bonds was observed at Phe-179 and His-176 residues having bond distances of 2.04 and 1.97 Å, respectively (Figure 3A). Figures 3B,C show the docking complexes of albigenin and epiafzelechin against COX-2, respectively. In albigenin and epiafzelechin docking complexes, a couple of hydrophobic pockets and hydrogen bonds was observed at particular residues with appropriate binding distances. The standard drug clavazin was also considered for docking analysis to check its binding profile against COX-2. Two hydrogen bonds were observed at Asn-344 and Asn-506 residues (Figure 3D).

### 4.5 *In vivo* analysis

#### 4.5.1 Serum level of biochemical variables in Cd-treated rats

A significant improvement in the biochemical variables has been observed in rats receiving phytochemical therapy after Cd administration (Table 5). A significant reduction has been detected in rats receiving monotherapy of luteolin, epiafzelechin, and albigenin (groups C, D, and E) after stimulation with Cd. In contrast, the levels of biochemical variables were restored significantly (20.253 ± 4.08 IU/L, 6.26 ± 2.28 ng/L, 5.79 ± 2.19 pg/mL, 11.16 ± 1.66 ng/mL, 51.36 ± 5.377, 3.19 ± 1.187, and 3.36 ± 7.027, respectively) in rats receiving combination treatment of three plant extracts as compared to those of group B (131.35 ± 1.57 IU/L, 28.16 ± 1.89 ng/L, 20.19 ± 2.19 pg/mL, 97.17 ± 2.18 ng/mL, 151.16 ± 3.16 pg/mL, 6.38 ± 2.27 nmol/mL, and 6.19 ± 2.17 ng/mL, respectively). Moreover, the levels of ALT, HNE, 8-OHdG, TNF-α, IsoP-2α, MDA, and COX-2 were amended remarkably in rats receiving combinational therapy of phytocompounds extracted from plants (Tables 5, 6).

**TABLE 5:** Biochemical response of luteolin, epiafzelechin, and albigenin in rats stimulated with Cd.

Group	Mean ± SD (n = 10)						
	ALT (IU/L)	HNE (ng/L)	8-OHdG (pg/mL)	TNF-α (ng/mL)	IsoP-2α (pg/mL)	MDA (nmol/mL)	COX-2 (ng/mL)
**A**	16.67 ± 1.75′	15.6 ± 1.98	1.05 ± 0.10	16.25 ± 1.68	91.15 ± 1.13	0.89 ± 0.076	0.91 ± 0.013
**B**	131.35 ± 1.57	28.16 ± 1.89	20.19 ± 2.19	97.17 ± 2.18	151.16 ± 3.16	6.38 ± 2.27	6.19 ± 2.17
**C**	25.26 ± 7.26	22.39 ± 1.42	27.49 ± 3.88	55.39 ± 4.66	112.36 ± 8.18	3.19 ± 1.66	4.07 ± 0.19
**D**	31.24 ± 3.26	17.39 ± 2.29	17.79 ± 3.99	66.53 ± 6.19	88.16 ± 5.55	7.19 ± 1.19	5.19 ± 1.79
**E**	41.46 ± 3.24	12.99 ± 1.75	23.89 ± 1.28	62.76 ± 3.46	88.46 ± 4.16	5.16 ± 1.48	2.19 ± 0.116
**F**	24.16 ± 3.36	14.76 ± 3.16	9.99 ± 2.18	55.20 ± 1.56	37.46 ± 4.16	3.10 ± 2.18	3.48 ± 0.16
**G**	65.20 ± 4.19	13.46 ± 3.66	24.36 ± 4.79	17.99 ± 4.49	88.46 ± 3.24	5.59 ± 5.37	1.19 ± 0.498
**H**	41.66 ± 4.39	8.88 ± 3.31	9.59 ± 4.66	18.36 ± 6.16	42.23 ± 7.33	5.49 ± 4.10	2.19 ± 0.105
**I**	20.253 ± 4.08	6.26 ± 2.28	5.79 ± 2.19	11.16 ± 1.66	51.36 ± 5.377	3.19 ± 1.187	3.36 ± 7.027
**LSD (0.05)**	8.26	7.32	6.25	6.26	10.26	1.22	1.06
***p*-value**	0.0156	0.014	0.013	0.019	0.000	0.011	0.000

**TABLE 6:** Pearson’s correlation coefficients of different variables in liver tissues of rats stimulated with Cd.

Variable	ALT	4-HNE	8-OHdG	TNF-α	IsoP-2α	MDA	COX-2
ALT	1	0.453**	0.671**	0.583**	0.495**	0.602**	0.404*
4-HNE		1	0.607*	0.690**	0.529**	0.490**	0.346**
8-OHdG			1	0.654**	0.654**	0.555**	0.469**
TNF-α				1	0.632**	0.594**	0.649**
IsoP-2α					1	0.476**	0.678**
MDA						1	0.469**
COX-2							1

**, correlation is significant at the 0.01 level (two-tailed).

## 5 Discussion

In the present study, we designed novel drugs of three phytochemicals through *in-silico* approaches. There has been considerable focus on phytocompounds as anti-inflammatory agents that could be used to cure or prevent diseases (Liu et al., 2013). The molecular docking analyses were performed on phytocompounds (luteolin, epiafzelechin, and albigenin) against COX-2 to identify the anti-inflammatory properties. The selected three phytocompounds showed high binding and anti-inflammatory activity against COX-2 (Table 4). Previous research ensured the significance of active sites consisting of amino acid residues (His-90, Val-117, Arg-120, Arg-121, Val-350, Leu-353, Ser-354, Tyr-355, Tyr-356, Leu-360, Phe-382, Tyr-385, Trp-387, Trp-388, Arg-513, Phe-519, Met-523, Val-524, Glu-527, Ala-528, Ser-530, Leu-531, and Leu-532) by X-ray crystallography (Orlando et al., 2015). Moreover, another structural analysis study of COX-2 depicts some key residues (Ala-185, Phe-186, Phe-187, Ala-188, Gln-189, His-190, Thr-192, His-193, Gln-194, Phe-196, Thr-198, Asn-368, Leu-370, Tyr-371, His-372, Trp-373, His-374, Leu-376, Leu-377, Val-433, Ser-437, Gln-440, Tyr-490, Leu-493, and Leu-494) involved in the binding pocket conformation (Amaravani et al., 2012). The comparative results showed that most of the residues were common, which ensured the significance of our docking results. These residues might be involved in inhibiting the inflammation process (Figure 7). Overexpression of COX-2 is linked to inflammation and many pathophysiological conditions like cancer and neuronal diseases (Choi et al., 2009; Amaravani et al., 2012). The *in vivo* results of the present study demonstrated the role of three phytochemicals in attenuating the inflammatory process along with significant antioxidant properties by activating various proteolytic enzymes that repair DNA and protein (Table 6). Our *in silico* results showed that three phytocompounds possessed good chemoinformatic and ADMET profiles (Table 3). The molecular properties of small compounds always depend on LogP values, including bioavailability and permeability of the membrane.

Long-range transport of cadmium pollution primarily occurs through air and water, eventually leading to topsoil deposition and accumulation in plants (e.g., tobacco). Tobacco smoke is the leading cause of Cd revelation (Satarug and Moore, 2004). The immediate effect of the Cd revelation is oxidative stress. The antioxidant glutathione (GSH) mitigates adverse physio-metabolic effects and defends against Cd stress (Jung et al., 2021). GSH reduction is also pretentious for the DNA duplication, repair, differentiation, propagation, and apoptotic mechanism (Chatterjee, 2013). DNA methylation and cell cycle developments are controlled by several cellular factors that can be activated or inactivated by Cd administration. DNA production and cell propagation rely on cumulative Cd concentrations (Yang et al., 2004). It has been found that the overexpression of the *OLE1* gene is central to reducing oxidative stress induced by Cd, possibly through inhibiting lipid peroxidation and protecting the cytoplasmic membrane from damage in yeast (Ozturk et al., 2021). Exposure to toxic Cd levels triggers different RAS kinase pathways, expressively MAPK (Jonak et al., 2004). Cd persuades the overexpression of proto-oncogenes like c-fos, c-myc, and c-jun (Spruill et al., 2002). Cd revelation may lead to tumor growth and propagation through expression of these genes. The Myb-type transcription factor activates Cd-induced cyclin D and cyclin E in mammalian cells (Bertin and Averbeck, 2006). Cd increases the production of tumor necrosis factors (TNF-α), interleukin-10 (IL-10), and heme oxygenase-1(HO-1) (Weber et al., 2006; Lawal et al., 2015; Bonaventura et al., 2018).

Metallothioneins (MTs) are cysteine-rich, heavy metal-binding proteins that play a central role in essential trace element homeostasis and detoxification (Cobbett and Goldsbrough, 2002). Mammalian metallothionein can protect in contradiction of Cd injuries (López et al., 2003) as it makes complexes with free-Cd through cysteine deposition. Cd and oxidative stress triggered metallothionein production and its gene transcription along with specific heat shock proteins (HSPs) that influence the apoptosis pathways (Kusakabe et al., 2008). Metallothionein is a transporter protein; the Cd–MT complex enters into the blood and dysfunction the multiple organs of the body (Sabolić, 2006) through the activation of G-protein–metal coupled receptors present at the cell surface. It activates phospholipase C (PLC) (Gu et al., 2018), resulting in the formation of IP3 (inositol triphosphate) and DAG (diacylglycerol). At the same time, IP3 releases Ca^2+^ from the endoplasmic reticulum. Ca^2+^ activates calpain, which plays a significant role in apoptosis through the cleavage of BAX/BAK (Sobhan et al., 2013). BAX triggers cytochrome C release and apoptosis-inducing factor (AIF) from mitochondria to cytosol (Garrido et al., 2006). Calpain-1 activates caspase 9, which activates caspase-3 and induces apoptosis (Nakajima et al., 2014). Cell death results when an external signal activates a metabolic pathway (Compton, 1992). Physiological and pathological conditions trigger eukaryotic apoptosis, a different form of cell death (Arends and Wyllie, 1991).

**FIGURE 4:**
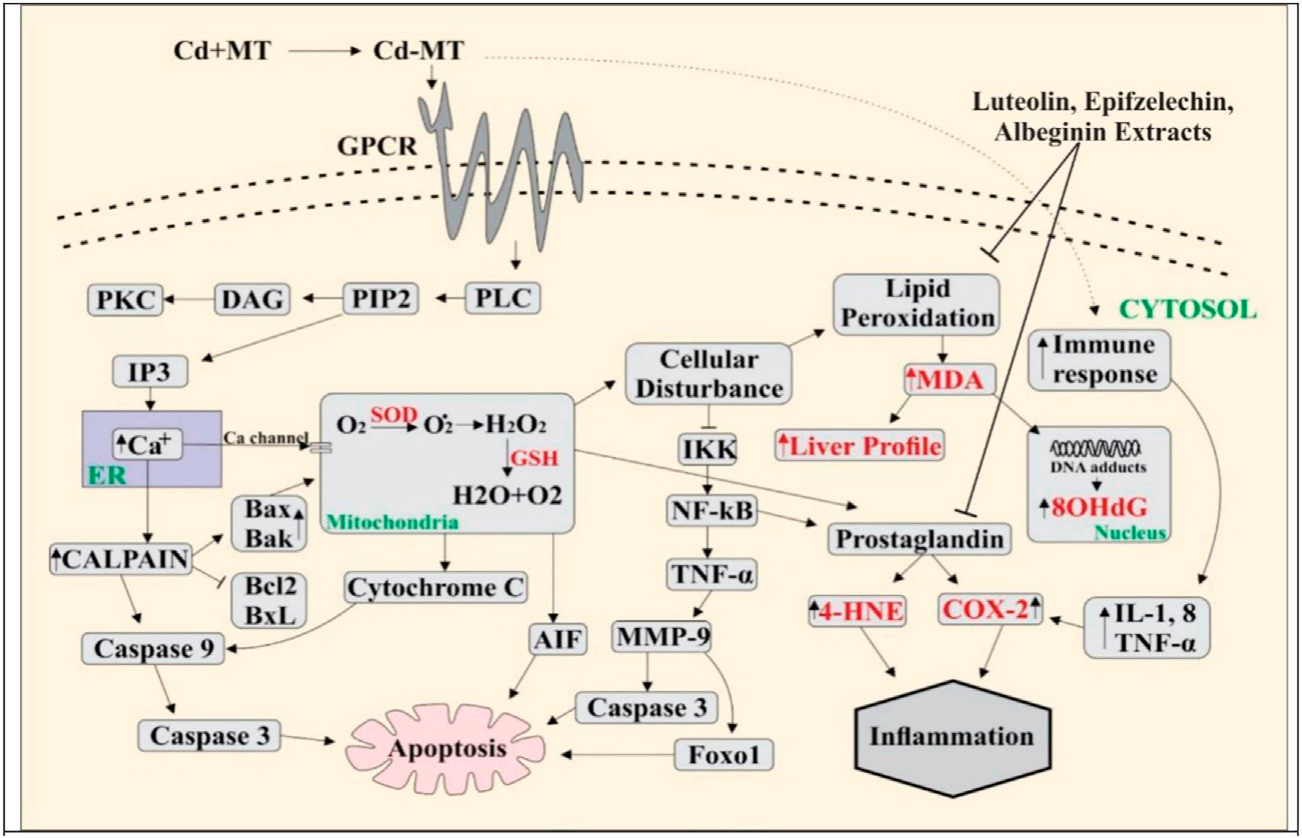
Cd enters blood by making a complex with metallothionein, and Cd binds with G-protein–metal coupled receptors on the cell surface, resulting in the activation of phospholipase C (PLC). Activated PLC results in PIP_2_ production that activates IP_3_, leading to the release of Ca^2+^ from ER. Ca^2+^ increases the level of calpain which further activates the caspases and as a result apoptosis occurs in the cell. Due to overloading, Cd enters into the mitochondria through the Ca^2+^ channels and ROS is produced in excess amount. This activates cytochrome C release and induces apoptosis through the caspases. Overproduction of ROS creates the disturbance in normal cell activity. Lipid peroxidation occurs when the level of MDA increases, DNA adducts are produced, and 8-OHdG levels are high in the nucleus. NF-kB is activated by the inhibition of IKK. NF-kB activates TNF-α, which produces MMP9, and apoptosis occurs through the activation of AKT and FOXO1. The PGE level increases by amplifying COX-2 and 4HNE. Immune response rises due to overloading Cd-activated inflammatory cytokines (IL-8, TNF, and IL-1), and the increase in the COX-2 level leads to inflammation.

The phenomenon of cell death is fundamental during multicellular growth to maintain homeostasis as it excludes undesirable cells (Vaux, 1993). Cd enters mitochondria either directly or through Ca^2+^, which results in increased levels of ROS through NADPH oxidase, which might be neutralized through the activation of the defense system by the cascade of reactions like SOD, CAT, and GSH (Smeets et al., 2009). Damaged epithelial cells formed the ROS with the activation of nicotinamide adenine dinucleotide phosphate (NADPH) oxidase (Fu et al., 2013). ROS produced in the mitochondrial cell through exogenous and endogenous signals promote prostaglandin production (Korbecki et al., 2013). Isoprostanes are free radical-catalyzed prostaglandin-like products of unsaturated fatty acids, such as arachidonic acid, eventually involved in inflammation and cardiovascular events (Halliwell and Lee, 2010; Bauer et al., 2014). In response to Cd, ROS can be formed by non-enzymatic and enzymatic reactions targeting the lung, liver, kidney, and testes following acute intoxication and causing nephrotoxicity, immunotoxicity, osteotoxicity, and tumors after prolonged exposure (Liu et al., 2009; Chmielowska-Bąk et al., 2014). Malondialdehyde (MDA) is the final product of lipid peroxidation and a relevant biomarker in clinical settings (Khoubnasab Jafari et al., 2015). MDA has a positive correlation with COX-2 (0.469**). Disorders in DNA occur due to the high MDA level, which is determined as 8-OHdG. Furthermore, the results of the present study showed that 8-OHdG has a positive correlation with MDA (0.555**). 8-OHdG is a stress biomarker and is a risk factor for cancer, diabetes, and atherosclerosis (Wu et al., 2004). Our study showed an 8-OHdG positive correlation with TNF-α (0.654**), IsoP-2α (0.654**), and COX-2 (0.469**). Owing to the disturbance in cells, I kappa B kinase (IKK) inhibits NF-kB, a nuclear factor pathway that takes part in inflammation. NF-kB is an inducible transcription factor for various procedures, like cell existence, differentiation, inflammation, and growth for genes, as shown in Figure 4.

In multiple systems, Cd activates NF-kB and TNF-α, further extending the inflammatory reaction. In the present study, TNF-α is positively correlated with IsoP-2α (0.632**) and MDA (0.594**). TNF-α increased the activation of matrix metalloproteinase (MMP), a protein involved in the degradation of the extracellular matrix of physiological and pathological processes (Kirschvink et al., 2005). MMP9 activates protein kinase B, which is involved in cell death, glucose metabolism, and cell proliferation (Endo et al., 2018; Ma et al., 2020). AKT is essential in activating caspase-3, which plays a role in cell death. MMP9 also activates Forkhead box protein O1 (FOXO1), which is critical for apoptosis. TNF-α increases cyclooxygenase 2 (COX-2) and 4-hydroxynonenal (4-HNE) levels, while COX-2 is a bifunctional enzyme that converts the arachidonic acid into prostaglandin and plays a vital role in inflammation. 4-HNE is vital for signal transduction, and it also plays a role in inflammation (Ayala et al., 2014; Hashim et al., 2022) and 4-HNE has a positive correlation with TNF-α (0.690**), IsoP-2α (0.529**), MDA (0.490**), and COX-2 (0.346**). The role of phytochemicals in Cd insult holds greater significance. A recent study showed that vitexin treatment reduced Cd-induced renal toxicity in rats (Umar Ijaz et al., 2021). Similarly, Hashim et al. (2022) found that *Catharanthus roseus* (CR) extract exhibited significant antioxidant activity against ROS-mediated DNA damage induced by Cd poisoning. The literature is in line with our findings that supports the use of bioactives for medicinal purposes.

Our findings provide evidence that the selected three phytocompounds ameliorated the toxic effects of Cd-induced toxicity by inhibiting inflammatory pathways. Hence, both *in silico* and *in vivo* data depicted the importance of these phytocompounds in novel drug design against inflammatory diseases.

## Data Availability

The original contributions presented in the study are included in the article/Supplementary Material; further inquiries can be directed to the corresponding author.
